# Proteomic analysis of the acquired enamel pellicle formed on human and bovine tooth: a study using the Bauru *in situ* pellicle model (BISPM)

**DOI:** 10.1590/1678-7757-2018-0113

**Published:** 2018-12-10

**Authors:** Vinícius Taioqui Pelá, Luiza Paula Silva Cassiano, Talita Mendes da Silva Ventura, Cintia Maria de Souza-e-Silva, Carlos Condarco Gironda, Daniela Rios, Marília Afonso Rabelo Buzalaf

**Affiliations:** 1 Universidade Federal de São Carlos, Departmento de Genetica e Evolução, São Carlos, SP, Brasil; 2 Faculdades Integradas Aparicio Carvalho, Porto Velho, RO, Brasil; 3 Universidade de São Paulo, Faculdade de Odontologia de Bauru, Departmento de Ciências Biológicas, Bauru, SP, Brasil

**Keywords:** Proteins, Saliva

## Abstract

The acquired enamel pellicle (AEP) is an organic film, bacteria-free, formed *in vivo* as a result of the selective adsorption of salivary proteins and glycoproteins to the solid surfaces exposed to the oral environment. Objective: This study aimed to compare the proteomic profile of AEP formed *in situ* on human and bovine enamel using a new intraoral device (Bauru *in situ* pellicle model - BISPM). Material and Methods: One hundred and eight samples of human and bovine enamel were prepared (4×4 mm). Nine subjects with good oral conditions wore a removable jaw appliance (BISPM) with 6 slabs of each substrate randomly allocated. The AEP was formed during the morning, for 120 minutes, and collected with an electrode filter paper soaked in 3% citric acid. This procedure was conducted in triplicate and the pellicle collected was processed for analysis by LC-ESI-MS/MS. The obtained mass spectrometry MS/MS spectra were searched against human protein database (SWISS-PROT). Results: The use of BISPM allowed the collection of enough proteins amount for proper analysis. A total of 51 proteins were found in the AEP collected from the substrates. Among them, 15 were common to both groups, 14 were exclusive of the bovine enamel, and 22 were exclusive of the human enamel. Proteins typically found in the AEP were identified, such as Histatin-1, Ig alpha-1, Ig alpha 2, Lysozyme C, Statherin and Submaxillary gland androgen-regulated protein 3B. Proteins not previously described in the AEP, such as metabolism, cell signaling, cell adhesion, cell division, transport, protein synthesis and degradation were also identified. Conclusion: These results demonstrate that the proteins typically found in the AEP appeared in both groups, regardless the substrate. The BISPM revealed to be a good device to be used in studies involving proteomic analysis of the AEP.

## Introduction

Teeth are constantly bathed by constituents from the gingival fluid, bacterial products, and by saliva. These constituents are rich in proteins and glycoproteins. As a result of this exposure, a bacteria-free organic film, known as acquired enamel pellicle (AEP), is formed ^1^ . The AEP formation is quick. Scanning electron microscopy showed that AEP can be detected even one minute after the enamel samples are exposed to the oral cavity ^2^ . Moreover, another *In vivo* study using proteomic approaches detected the presence of 89 proteins within the AEP, formed 5 minutes after dental prophylaxis ^3^ , while a recent study identified 190 proteins within the acquired pellicle, formed *In situ* for 3 minutes on ceramic specimens ^4^ .

The protection of the tooth surface by the AEP is well established in the literature and has been demonstrated in several studies. The AEP acts as a diffusion barrier or permeable membrane, diminishing the direct contact between the acids and the tooth surface, thus reducing the dissolution rate of hydroxyapatite ^5^ ^-^ ^10^ . *In vitro* studies revealed that the first proteins to electrostatically interact with the enamel surface are proline-rich proteins (PRPs), statherin and histatins ^11^ ^,^ ^12^ , while *In vivo* experiments revealed also the presence of mucins, amylase, cystatin, lysozyme and lactoferrin in the very initial stages of pellicle formation ^3^ ^,^ ^13^ .

The comprehension of the AEP protein profile was greatly increased with the advent of proteomic tools. Most of the AEP proteomic studies available so far were conducted *In vivo* ^3^ ^,^ ^10^ ^,^ ^13^ ^-^ ^16^ . While the *in vivo* model provides the most clinically relevant information, in some cases it cannot be used. One of them is when it is desirable to know the protein composition of the acquired pellicle formed on mixed surfaces constituted by teeth and restorative materials. Another situation is when it is necessary to evaluate the protein composition of the acquired pellicle formed on dentin surfaces ^17^ , considering it is quite difficult to obtain exposed dentin surfaces in an extent that allows the collection of material to be analyzed. In these situations, *In situ* models are desirable and to the best of our knowledge only two studies are available in the literature so far ^4^ ^,^ ^17^ . In the study by Delecrode, et al. ^17^ (2015), human root dentin specimens were used in a palatal appliance. One of the main limitations of the study was the fact that only a few typical proteins of the acquired pellicle were identified. As for the study by Delius, et al. ^4^ (2017), the authors employed ceramic specimens. Despite they were able to identify more than 100 proteins, some of which are typically found in the AEP ^4^ , the composition of the ceramic specimens is quite different of human enamel, which certainly impacts in the protein profile of the acquired pellicle.

In studies involving dental caries and erosion, bovine teeth, which are easier to obtain, are often used as surrogate for human teeth ^18^ ^-^ ^20^ . However, there are no studies comparing the protein profile of the AEP formed on human and bovine specimens. Considering that structural differences between these two types of substrates exist ^21^ , with bovine crystallites being thicker ^22^ and bovine enamel presenting higher radiographic density ^23^ than the human counterparts, there could be differences in the protein profile of the AEPs formed on these two types of substrates. In addition, one of the main limitations of studies involving proteomic analysis of the acquired pellicle is to obtain enough material to be analyzed. Thus, it is of great interest to develop devices to be used in *in situ* studies that make possible the collection of appropriate amounts of AEP to allow proper protein identification and quantification in proteomic studies. Therefore, this study aimed to compare the proteomic profile of the acquired enamel pellicle (AEP) formed *in situ* on human and bovine enamel using a new device (Bauru *in situ* pellicle model - BISPM), especially designed to allow the collection of enough amount of AEP to be analyzed using proteomic approaches.

## Material and methods

### Preparation of bovine and human specimens

Bovine permanent incisors and third human molar were recently extracted, disinfected and kept in 0.1% buffered thymol solution (pH 7.0). After 30 days, a visual inspection was conducted to evaluate the presence of caries, stains and cracks. In these cases, the teeth were excluded.

The selected teeth (n=108) had their crowns cut (4×4×2 mm) using a precision cutting machine (ISOMET Low Speed Saw Buehler Ltda., Lake Bluff, Illinois, USA) with two diamond discs (double-sided XL 12205 ‘high concentration’, 102×12.7×0.3 mm^3^; Extec Diamont Wafering Blade®, Enfield, Connecticut, USA) attached. After that, only the dentin of the specimens was ground flat with water-cooled silicon carbide discs (320 grade of Al_2_O_3_ paper; Buehler). Then, the specimens were kept with wet gauze in a refrigerator at 4°C prior to the experiment.

### Ethical aspects and subjects

The local Ethics Committee approved the protocol of this research (no. 58331216.9.0000.5417; Ethics Committee of the Bauru School of Dentistry, University of São Paulo), which followed the guidelines of good clinical practice and conformed to the Declaration of Helsinki. Nine young adult volunteers of both genders took part in the study after signing an informed consent document. The sample size was chosen based on results of previous studies ^10^ ^,^ ^14^ ^,^ ^17^ . The exclusion criteria were: smokers, presence of caries lesions, gingivitis, periodontitis, low salivary flow (unstimulated and stimulated to be greater than 0.3 and 1.0 mL/ minute, respectively) and the use of medicines that could change the salivary composition or flow-rate.

### Bauru *in situ* pellicle model (BISPM)

**Figure 1:**
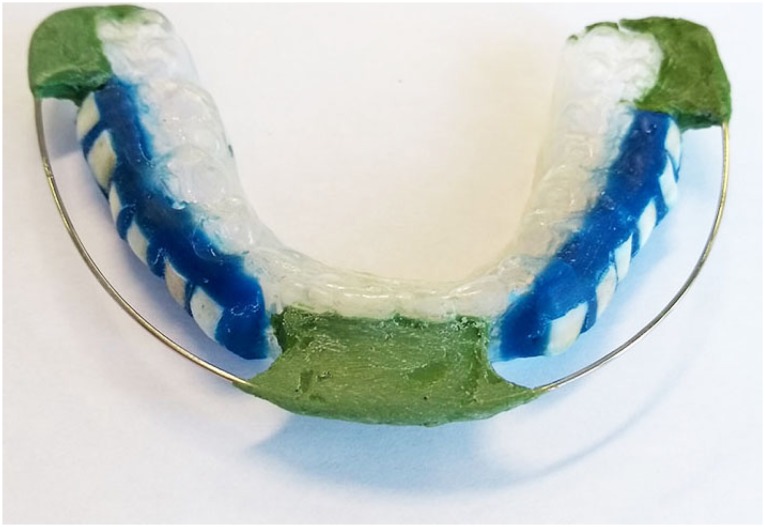
Bauru *in situ* pellicle model (BISPM)

Alginate impressions were used to make plaster models, employed to prepare silicon devices from the mandibular arches of each volunteer. The enamel samples were cleaned in an ultrasound (T7 Thornton, a Unique Ind. e Com. Ltda., São Paulo, SP, Brazil) for 7 minutes at 25°C. Twelve specimens (6 from each group) were placed in the recessed sites and fixed with dental wax (Asfer Indústria Química Ltda®, São Caetano do Sul, SP, Brazil) for the formation of the AEP *in situ.* A number 0.8 orthodontic wire (Morelli Ortodontia^®^, Sorocaba, SP, Brazil) was suspended above the specimens ^24^ , to avoid direct contact of the mucosa with them, thus preventing the impact of mechanical forces on AEP formation. This wire was fixed on the silicon devices with godiva (Kerr Corporation^®^, Orange, Califórnia, USA) in the center of the apparatus and in the posterior regions ( Figure 1 ).

### *In situ* experiment

The experiment was conducted during the morning to abstain from circadian effects on the composition of the pellicle ^16^ for 3 consecutive days, aiming to obtain enough material to be submitted to the proteomic analysis. Firstly, the volunteers inserted the intraoral device into their mouths and for 120 minutes they were instructed not to eat or drink to allow the AEP to form on the enamel surfaces. For the AEP collection, the intraoral device was removed from the mouth and the samples were washed with deionized water then dried by air. An electrode filter paper 5×10 mm (Electrode Wick, Bio-Rad^®^, Hercules, Califórnia, USA) pre-soaked in 3% citric acid ^10^ was rubbed on the surface of the enamel samples with the aid of tweezers to collect the AEP. Twelve strips were used for each participant. The filter papers were stored separately for each group in a polypropylene microcentrifuge tube at −80°C until the proteomic analyses.

### Preparation of the AEP samples

The AEP samples were prepared for proteomic analysis according to a recently standardized protocol ^15^ . Briefly, protein extraction was performed twice using a solution containing 6 M urea, 2 M thiourea in 50 mM NH_4_HCO_3_ pH 7.8, and the supernatants were stored. To increase protein recovery, the wick papers were transferred to filter tubes (Corning CostarSpin-X Plastic Centrifuge Tube Filters^®^, SigmaAldrich, New York, New York, USA), centrifuged, and the supernatant was collected. The supernatants were pooled, centrifuged again and transferred to a falcon tube. Then, 50 mM NH_4_HCO_3_ were added to dilute the urea and thiourea, and the samples were placed in Falcon Amicon tubes (Amicon Ultra – 15 Centrifugal Filter Units - Merck Millipore^®^, Tullagreen, County Cork, IE), centrifuged and concentrated to approximately 150 μL. Reduction [5 mM dithiothreitol (DTT) for 40 minutes at 37°C] and alkylation [10 mM iodoacetamide (IAA) in the absence of light for 30 minutes] were performed. Samples were then digested using 2% (w/w) trypsin (Promega^®^, Madison, Wisconsin, USA). Then 10 μL of 5% formic acid was placed to stop the action of trypsin. C18 Spin columns (Thermo Scientific^®^, Rockford, Illinois, USA) were used to desalt and purify the samples, and protein was quantified using the Bradford method (Bio-Rad^®^, Hercules, Califórnia, USA). The amount of protein obtained was 14.06 and 21.81 μg for bovine and human groups, respectively. The samples were resuspended in a solution containing 3% acetonitrile and 0.1% formic acid to be submitted to nano LC-ESI-MS/MS.

### Shotgun label-free quantitative proteomic analysis

Peptides identification was performed as previously described ^15^ on a nanoACQUITY UPLC-Xevo QTof MS system (Waters, Manchester, New Hampshire UK). The nanoACQUITY UPLC was equipped with nanoACQUITY HSS T3, analytical reverse phase column (75 μm x 150 mm, 1.8 μm particle size, Waters).

ProteinLynx Global Server (PLGS) version 3.0 (Waters Co., Manchester, New Hampshire, UK) was used to process and search the continuum LC-MSE data. Proteins were identified with the embedded ion accounting algorithm in the software and a search of the *Homo sapiens* database (reviewed only, UniProtKB/ Swiss-Prot), downloaded on June 2015 from UniProtKB ( http://www.uniprot.org/ ). The identified proteins were classified and assigned by biological function ^16^ ^,^ ^25^ , origin and molecular interaction ( http://www.uniprot.org/ ).

For label-free quantitative proteome, three MS raw files from each pooled group were analysed using the PLGS software. All the proteins identified with confidence score greater than 95% were included in the quantitative analysis. Identical peptides from each triplicate by sample were grouped based on mass accuracy (<10 ppm) and on time of retention tolerance <0.25 min, using the clustering software embedded in the PLGS. Difference in expression among the groups was expressed as p<0.05 for down-regulated proteins and 1-p>0.95 for up-regulated proteins.

## Results

**Figure 2:**
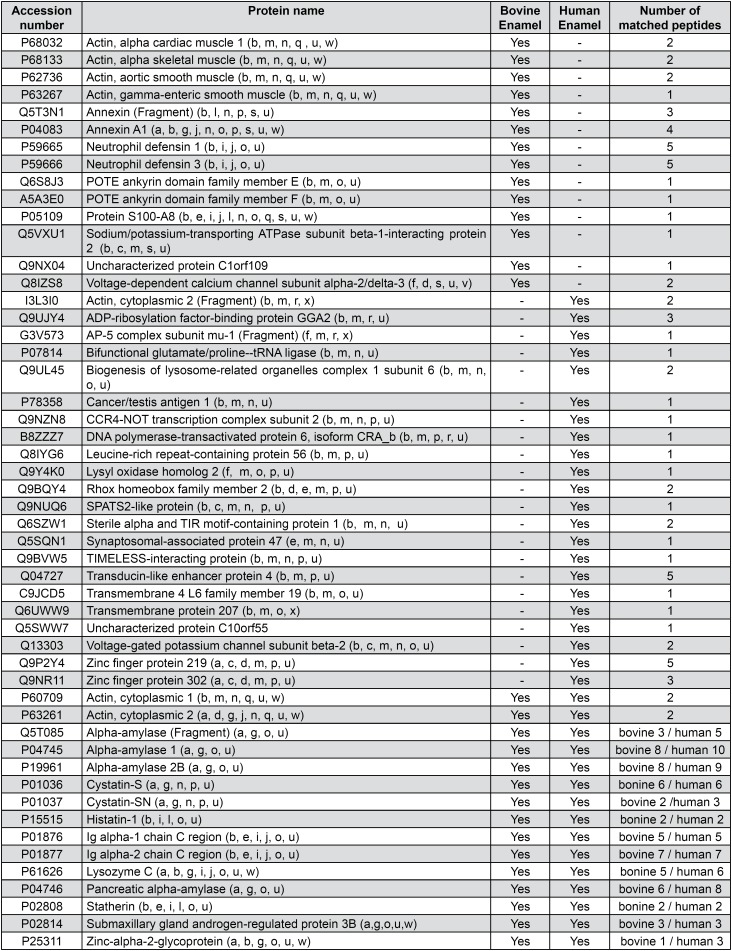
Total number of identified proteins, considering both substrates

**Figure 3:**
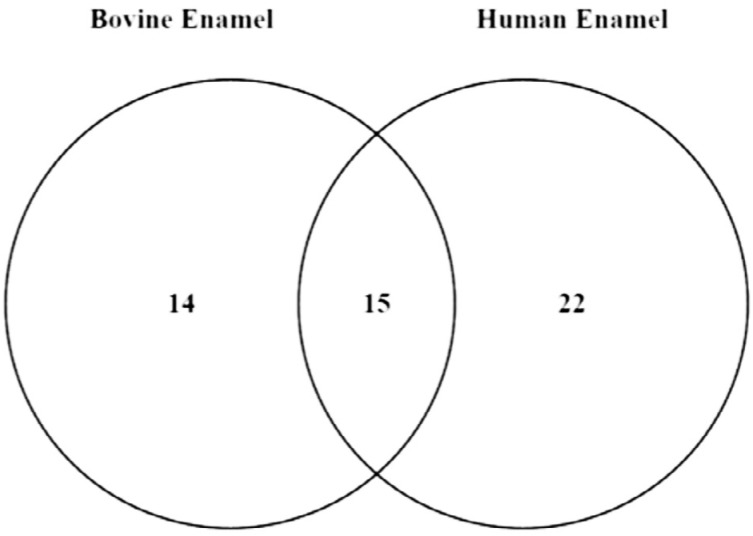
Venn Diagram with the numbers of the exclusive proteins from each group and the common proteins between two groups

The use of BISPM allowed the collection of enough proteins for proper analysis. The total number of identified proteins, considering both substrates, was 51. These proteins were classified according to their biological function, origin and molecular interaction ( Figure 2 ). Considering these proteins, 15 were common for both groups, 14 were present only on the bovine enamel group, and 22 proteins were exclusive of the human enamel ( Figure 3 ).

The proteins found in both groups ( Figure 2 ) are typical components of the AEP, such as isoforms of Cystatin, Actin, Alpha-amylase and Ig A, Histatin 1, Lysozyme C, Statherin, Submaxillary gland androgen-regulated protein 3B and Alpha-amylase. Zinc-alpha- 2-glycoprotein, involved in the detection of chemical stimulus and in sensory perception of bitter taste, was also identified in both groups.

Most of the proteins found exclusively in the AEP collected from the bovine enamel are not typically described among the constituents of the AEP and are related to cell motility (distinct isoforms of actin), immune response (Annexin A1 and distinct isoforms of neutrophil defensin), as well as binding to calcium (Protein S-100-A8 and Voltage-dependent calcium channel subunit alpha-2/delta-3) ( Figure 2 ). As for the proteins identified uniquely in the AEP collected from human enamel, most of them are not commonly described intracellular proteins and with unknown function in the AEP ( Figure 2 ). These proteins might have been originated from the oral mucosa.

Regarding the quantitative analysis, two proteins increased in human enamel, when compared to bovine enamel (Lysozyme C and Pancreatic alpha-amylase), while two isoforms of Actin cytoplasmic, Histatin 1 and Statherin decreased ( Table 1 ).

**Table 1:** Classification and relative quantification of proteins identified in the acquired enamel pellicle collected from Human Enamel (HE) and Bovine Enamel (BE)

Accession number	Protein name	Ratio HE/BE	p
P61626	Lysozyme C	1,1	1
P04746	Pancreatic alpha-amylase	1,18	0.99
P60709	Actin, cytoplasmic 1	0,77	0.03
P63261	Actin, cytoplasmic 2	0,76	0
P15515	Histatin-1	0,67	0
P02808	Statherin	0,73	0

## Discussion

The main challenge in studies involving proteomic of the AEP is to obtain enough protein to allow proper analysis. To overcome this difficulty, we developed a new device, the BISPM that has a special design in order to optimize the collection of enough proteins from the AEP. The placement of number 0.8 orthodontics wire suspended above the specimens to avoid direct contact of the mucosa with them ^24^ was the main responsible for it. In addition, the experiment was carried out in 3 consecutive days, and the samples collected from the same treatments were pooled. Furthermore, we worked with a mandibular apparatus instead of a palatal one, because more saliva is expected to bath the specimens in the first condition due to the gravity force. These strategies were effective to allow enough proteins in the AEP to be analysed. However, i t has been shown that the composition of the AEP changes according to its location in the dental arches ^15^ , which is a limitation of our model, despite a recent study revealed no difference in the protective ability against initial erosion of the AEP formed *In situ* using palatal and mandibular intraoral device ^24^ . It is worth mentioning that the removable apparatus developed was well tolerated by the volunteers, without any reported discomfort.

In this study, the total number of identified proteins, when both substrates are considered, was 51. This is quite similar to the number of proteins identified in an experiment where the pellicle was collected from dentin specimens *In situ* using a mandibular device ^17^ . In the study by Delecrode, et al. ^17^ (2015), the protocol for collecting the acquired pellicle formed on the dentin specimens was similar to the one employed in this study, considering that this protocol is widely used in *In vivo* experiments ^10^ . Despite the similar number of proteins identified, the pattern of proteins in the study by Delecrode, et al. ^17^ (2015) was very different from ours. The only protein typically described in the acquired pellicle was mucin ^17^ , while in this study we were able to identify many typical proteins, such as isoforms of Cystatin, Actin, Alpha-amylase and Ig A, Histatin 1, Lysozyme C, Statherin, Submaxillary gland androgen-regulated protein 3B and Alpha-amylase. These differences might be explained due to the distinct types of substrates (dentin X enamel).

In a recent study, the authors collected the acquired pellicle formed for 3 min on ceramic specimens *In situ* and a total of 190 proteins were identified ^4^ , among which 58% have been described in the pellicle before ^4^ and some of them were also identified in this study, such as Lysozyme C, IgA, Protein S-100, Cystatins, Neutrophil defensin and Alpha amylase. The higher number of identified proteins might be explained due to the high total surface area of the specimens [8×3 cm ^2^ in this study and in the one by Delecrode, et al. ^17^ (2015)], as well as to the protocol of collection of the acquired pellicle. As mentioned above, in this study and in the study by Delecrode, et al. ^17^ (2015), the pellicle was collected with wick filters embedded in 3% citric acid, while in Delius, et al. ^4^ (2017) the pellicle proteins were eluted by incubation in TRIS-HCl buffer containing SDS, followed by ultrasonication in RIPA-buffer, which cannot be done *In vivo.* Moreover, despite the ceramic used, Delius, et al. ^4^ (2017) presents protein adhesion forces close to those of hydroxyapatite, and both substrates share common isoelectric points ^26^ . The result of using natural teeth certainly is much closer to the clinical condition.

This study aimed to compare the protein composition of the acquired pellicle formed on bovine and human enamel. Bovine enamel has been used as surrogate for human enamel dental research. There are publications comparing both substrates regarding chemical composition, physical properties, dental caries, dental erosion/abrasion, bonding and microleakage studies ^27^ , however, the composition of the acquired pellicle formed on both substrates has never been compared. Bovine teeth are much easier to obtain than human teeth and are also bigger, thus providing specimens with a higher surface area, which is a desirable characteristic for studies involving collection of acquired pellicle. In this study, despite some differences found in the proteome of the acquired pellicle formed on bovine and human enamel, most of the proteins typically found in the AEP were present in both substrates ( Figure 2 ), without significant fold change for most of them. This might be due to the similar inorganic composition of bovine and human enamel (bovine enamel has only 1% more calcium content in weight when compared with human enamel; and the carbonate concentrations are quite similar in both substrates ^27^ ). Moreover, the crystallographic nanoscale structure of bovine enamel is very similar to human enamel ^28^ . It should be noted that the precursor proteins that constitute the first layers of the AEP are those with affinity to hydroxyapatite ^29^ . These results indicate that bovine enamel can be used as a substitute for human enamel in *In situ* studies involving proteomic analysis of the AEP. Moreover, the BISPM seems to be an appropriate device to be used in such studies.
